# Pharmacogenomics of Leukotriene Modifiers: A Systematic Review and Meta-Analysis

**DOI:** 10.3390/jpm12071068

**Published:** 2022-06-29

**Authors:** Yuxuan Zhao, Xinyi Zhang, Congxiao Han, Yuchun Cai, Sicong Li, Xiaowen Hu, Caiying Wu, Xiaodong Guan, Christine Lu, Xiaoyan Nie

**Affiliations:** 1 Department of Pharmacy Administration and Clinical Pharmacy, School of Pharmaceutical Sciences, Peking University, Beijing 100191, China; 2011210066@stu.pku.edu.cn (Y.Z.); 18782201139@163.com (X.Z.); han_congxiao@126.com (C.H.); cyctiancheng@pku.edu.cn (Y.C.); lisicong32@163.com (S.L.); 2111210044@stu.pku.edu.cn (X.H.); 13413682884@163.com (C.W.); guanxiaodong@pku.edu.cn (X.G.); 2 Department of Population Medicine, Harvard Medical School and Harvard Pilgrim Health Care Institute, Boston, MA 02115, USA; christine_lu@hphci.harvard.edu

**Keywords:** asthma, personalized medicine, pharmacogenetics, leukotriene receptor antagonist

## Abstract

Pharmacogenetics research on leukotriene modifiers (LTMs) for asthma has been developing rapidly, although pharmacogenetic testing for LTMs is not yet used in clinical practice. We performed a systematic review and meta-analysis on the impact of pharmacogenomics on LTMs response. Studies published until May 2022 were searched using PubMed, EMBASE, and Cochrane databases. Pharmacogenomics/genetics studies of patients with asthma using LTMs with or without other anti-asthmatic drugs were included. Statistical tests of the meta-analysis were performed with Review Manager (Revman, version 5.4, The Cochrane Collaboration, Copenhagen, Denmark) and R language and environment for statistical computing (version 4.1.0 for Windows, R Core Team, Vienna, Austria) software. In total, 31 studies with 8084 participants were included in the systematic review and five studies were also used to perform the meta-analysis. Two included studies were genome-wide association studies (GWAS), which showed different results. Furthermore, none of the SNPs investigated in candidate gene studies were identified in GWAS. In candidate gene studies, the most widely studied SNPs were ALOX5 (tandem repeats of the Sp1-binding domain and rs2115819), LTC4S-444A/C (rs730012), and SLCO2B1 (rs12422149), with relatively inconsistent conclusions. LTC4S-444A/C polymorphism did not show a significant effect in our meta-analysis (AA vs. AC (or AC + CC): −0.06, 95%CI: −0.16 to 0.05, *p* = 0.31). AA homozygotes had smaller improvements in parameters pertaining to lung functions (−0.14, 95%CI: −0.23 to −0.05, *p* = 0.002) in a subgroup of patients with non-selective CysLT receptor antagonists and patients without inhaled corticosteroids (ICS) (−0.11, 95%CI: −0.14 to −0.08, *p* < 0.00001), but not in other subgroups. Variability exists in the pharmacogenomics of LTMs treatment response. Our meta-analysis and systematic review found that LTC4S-444A/C may influence the treatment response of patients taking non-selective CysLT receptor antagonists for asthma, and patients taking LTMs not in combination with ICS for asthma. Future studies are needed to validate the pharmacogenomic influence on LTMs response.

## 1. Introduction

Asthma is a common disease, affecting 1–18% of the child population and 0.2–21.0% of the adults in different countries, and could be characterized by chronic airway inflammation, airway hyperresponsiveness, and airway obstruction [1,2]. It is accompanied by cough, wheeze, shortness of breath, and chest tightness that vary overtime and in intensity. Inhaled corticosteroids (ICS), leukotriene modifiers (LTMs), and β_2_-adrenergic receptor agonists are typically used medications for asthma. However, patients with asthma vary in disease onset, exacerbating stimuli, severity, and response to these medications [3]. It is reported that these heterogeneities could stem from disease history, comorbidities, exposure to allergens, medication adherence, and gene polymorphism. For drug response, it could be influenced by age, gender, renal function and excretory capacity, liver function and metabolic capacity, comorbidity, pregnancy, drug-food interactions, drug-drug interactions, and genetic factors [4]. Among these factors, existing research estimated that genetic variation may result in 60% to 80% of the variability in therapeutic response to anti-asthmatic drugs [5].

Recent research has demonstrated that leukotrienes (LTs), a series of inflammatory cytokines, play an important role in the pathogenesis of asthma. LTs are generated by the metabolism of arachidonic acid (AA) through the 5-lipoxygenase (5-LO) metabolism pathway [6,7,8,9]. AA is metabolized to produce LTA4, which is in turn converted into LTB4 or LTC4. LTC4 will then be transported extracellularly and catalyzed to form LTD4 and LTE4. These LTs bind to cognate receptors to contract the bronchial smooth muscle, promote the aggregation of inflammatory cells, and so forth. Thus, restraining LTs through blocking receptors or obstructing metabolic pathways (including leukotriene receptor antagonists (LTRA) like montelukast and 5-lipoxygenase inhibitors like zileuton) is an important way to treat asthma. LTMs are recommended as the controller for mild to moderate asthmatics, and long-acting β_2_-agonists (LABA) in combination with ICS are recommended as the alternative regimen for moderate to severe asthmatics, especially for children [1]. Response to asthma treatments has been evaluated by the provocative concentration of methacholine and a subsequent 20% drop in FEV_1_ (PC_20_), forced expiratory volume in 1 s (FEV_1_), the peak expiratory flow rate (PEFR), number of puffs of short-acting β_2_-agonist per day, and number of asthma exacerbations. Studies conducted in children tended to focus on asthma exacerbations while studies in adults usually assess lung function as the primary outcome.

According to the National Institutes of Health (NIH), pharmacogenomics is a novel field that combines pharmacology and genomics to explore how genes affect an individual’s response to drugs [10]. Most studies have focused on ICS and β_2_-agonists, but evidence on LTMs is accumulating. 

In light of widely varying LTM treatment responses and the important role of polymorphism in patients with asthma, we systematically reviewed the pharmacogenetic literature on LTM and performed a meta-analysis on LTC4S-444A/C gene polymorphism.

## 2. Materials and Methods

We searched exhaustively in PubMed, EMBASE, and Cochrane using specified search terms (Box 1) for pharmacogenetic studies on LTM published until May 2022. Conference abstracts and studies that were not conducted in humans were excluded. The association between gene polymorphism and drug response was analyzed from the perspective of different genes in this study. The meta-analysis has been registered in PROSPERO (ID: CRD42021258332), and no amendments have been made to the information provided at registration or in the protocol [11]. Reporting of this paper follows the PRISMA guidance (Table S1) [12]. 

Keywords, titles, and abstracts were screened to determine whether studies investigated the association between specific genetic polymorphisms and response to LTM. Any duplicates were immediately removed at this stage. Remaining studies then proceeded to full-text review. We also searched references of these studies to identify any other eligible studies. Eventually, a total 31 studies were included in our systematic review. Studies that were included in the systematic review and studied LTC4S-444A/C polymorphism in asthma patients were selected for meta-analysis. The inclusion criteria of the meta-analysis were as follows: (1) explored the association between LTC4S-444A/C polymorphism and lung function, (2) provided sufficient data (sample size, change or improvement of lung function, 95% confidence interval or standard deviation) available to calculate the effect size, and (3) showed genotype distributions in the original studies conforming to Hardy–Weinberg equilibrium (HWE). The following data of all studies included in the systematic review and meta-analysis were extracted: year of publication, author, outcome, the definition of the outcome, genes, single nucleotide polymorphisms (SNPs), medication type, dosage regimen, drug combination, study population, race, age range, sample size, and follow-up time. Two reviewers independently extracted the data. Any disagreement concerning data extraction was settled through consensus between authors. Quality assessment was performed by Newcastle–Ottawa Scale (NOS).

Box 1 **Search term**

(("Asthma"[MeSH Terms] OR "Anti-Asthmatic Agents"[MeSH Terms]) OR (asthmatic*)) AND ((((((("Lipoxygenase Inhibitors"[Mesh] OR "Lipoxygenase Inhibitors" [Pharmacological Action]) OR (“Leukotriene Antagonists"[Mesh] OR "Leukotriene Antagonists" [Pharmacological Action])) OR "montelukast" [Supplementary Concept]) OR "zafirlukast" [Supplementary Concept]) OR "pranlukast" [Supplementary Concept]) OR "zileuton" [Supplementary Concept])

AND

(((("Pharmacogenetics"[Mesh]) OR "Polymorphism, Genetic"[Mesh]) OR "Genome-Wide Association Study"[Mesh]) OR "Genetic Association Studies"[Mesh] (((("Pharmacogenetics"[Mesh]) OR "Polymorphism, Genetic"[Mesh]) OR "Genome-Wide Association Study"[Mesh]) OR "Genetic Association Studies"[Mesh]) OR (candidate genes)))

We analyzed the data using Revman 5.4 and R-4.1.0 software. The primary outcome was analyzed by the dominant model, i.e., AA vs. AC (or AC + CC) genotype. Meta-analysis was performed by combining each study’s results using the inverse variance statistical method and the random effects model. The strength of the association between LTC4S-444A/C polymorphism and improvement of lung function parameters was evaluated by mean difference (MD) with 95% confidence intervals (CI) and *p*-value. The significance of MD was determined by Z-test, and a *p*-value less than 0.05 was considered statistically significant. Heterogeneity was assessed by I^2^ statistic and χ^2^ test. I^2^ value greater than 50% was considered as the signal of heterogeneity. In cases of heterogeneity, subgroup analyses were conducted to find the source of heterogeneity, and sensitivity analyses that excluded a single study each time were used to test the stability of results. Subgroup analyses were performed by ethnicity, asthma severity, types of drugs, concomitant medication, and follow-time. The change in significance of *p*-value in sensitivity analyses indicates that the results may be unstable. In addition, we used Pearson’s χ^2^ test to examine HWE in each study included, and for *p*-values greater than 0.05 it was considered that the genotype distribution among participants was in HWE. The quality of each study was evaluated by Newcastle–Ottawa Scale (NOS), which included aspects of selection, comparability, and exposure, and a score less than 5 was considered unacceptable. The possible publication bias was investigated using Begg’s funnel plot and Egger’s test, and funnel plot asymmetry and *p*-value less than 0.05 were considered as indicators of publication bias.

## 3. Results

**Figure 1:**
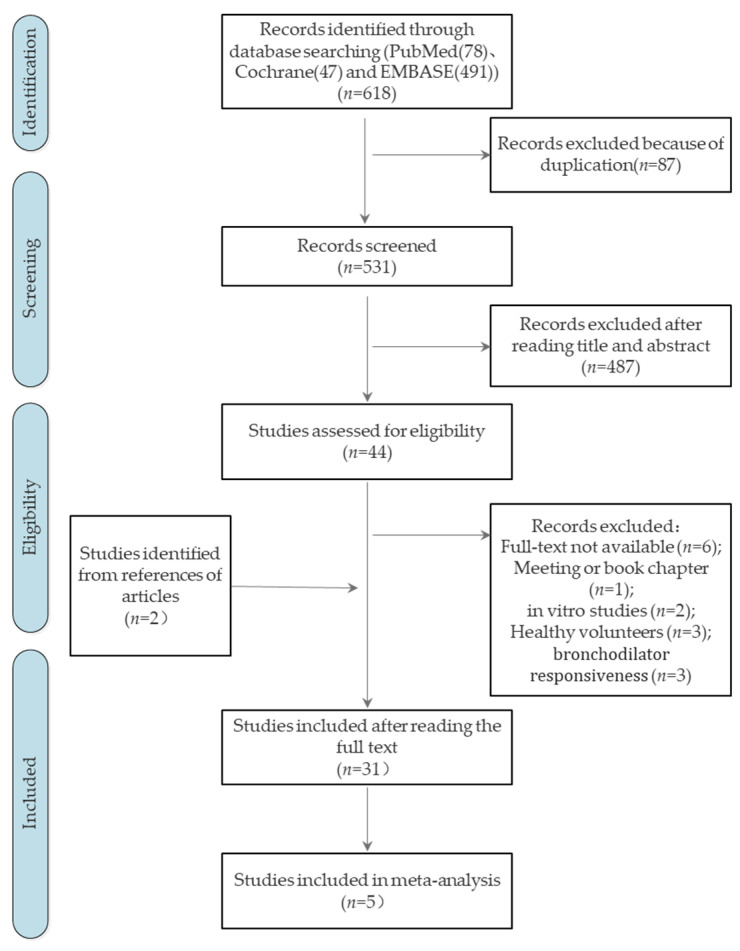
Screening process.

We identified 80 cohort or case-control studies in PubMed, 491 in EMBASE, and 47 in Cochrane. The screening process for this systematic review is shown in Figure 1. Finally, 31 studies with 8084 participants were included in our systematic review (Table 1).

After screening titles and abstracts of studies included in this systematic review, we identified 14 studies with 873 participants related to LTC4S-444A/C polymorphisms. Five studies on the association between LT4CS-444A/C polymorphisms and improvement of lung function parameters were eventually included in the meta-analysis. Due to the limited number of current studies, we included all measures of improvement of lung function parameters. Considering the lack of patients with the CC genotype, we aggregated AC and CC genotypes and compared them to the AA genotype.

### 3.1. ALOX5

ALOX5 encodes lipoxygenase which catalyzes the conversion of arachidonic acid to leukotriene A4. Leukotrienes play an important role in inflammatory conditions. Two of the most studied SNPs about ALOX5 were ALOX5 (tandem repeats of the Sp1-binding domain) and ALOX5 (rs2115819). ALOX5 (tandem repeats of the Sp1-binding domain) is likely to influence asthma exacerbations in patients taking LTMs [13,14,15,16,17]. Lima et al. found variant allele (either 2, 3, 4, 6, or 7 repeat allele) is associated with lower risk of asthma exacerbations [13]. However, Nwokoro et al. found an opposite result [14]. Telleria et al. found that patients with at least one five repeat allele had lower exacerbations rate, better FEV_1_ response and less β_2_ rescue medication [15]. Similar effects of ALOX5 (tandem repeats of the Sp1-binding domain) on lung function in patients with asthma were also found in studies by Fowler et al. and Drazen et al. [16,17]. ALOX5 (rs2115819) is more likely to be associated with lung function after LTM treatment [13,18,19]. Lima and Tantisira et al. found that patients with GG homozygous had better FEV_1_ response compared with others in white adults [13,18]. Kotani et al. showed a similar result in Japanese adults [19]. A study investigated association between ALOX5 (rs4987105 and rs4986832) and lung function, obtaining a significant result [20] (Table 2).

### 3.2. LTA4H (rs2660845)

LTB4 is generated from LTA4 by leukotriene A4 hydrolase, which is present in high concentrations in asthmatic airways [44,45]. In addition, the mRNA expression of leukotriene A4 hydrolase is enhanced in asthmatics [45]. Current research suggests that, for the LTA4H (rs2660845), AA genotypes are probably associated with better clinical response to montelukast. Lima et al. found that white adult patients carrying G allele were more likely to have asthma exacerbations than patients with AA homozygous after 6 months of montelukast treatment [13]. Similarly, a significant association was observed in two European and one African Americans early-onset asthma cohorts, but not in Hispanics/Latinos early-onset asthma cohorts and Europeans late-onset asthma cohorts [21]. A meta-analysis conducted on the above cohorts obtained the same results as well. In terms of lung function, Kotani et al. found that changes in FEV_1-0_ and PEF from baseline significantly improved in Japanese adult patients with AA homozygous after montelukast intake [19]. AA genotype tended to have higher symptom scores, though this was not statistically significant.

### 3.3. LTC4S-444A/C (rs730012)

LTC4 synthase (LTC4S), is the endmost and sole committed enzyme in the generation of the proinflammatory mediators [46]. LTC4 is generated from LTA4 binding to reduce glutathione by the enzymatic action of LTC4S. In addition, immunohistochemical studies showed that LTC4S was overrepresented in aspirin-intolerant asthmatics (AIA) and associated with the overly high concentration of cysteinyl leukotrienes and lysine-aspirin bronchial hyperreactivity [47].

According to existing research, LTC4S-444A/C probably has no effect on lung function in asthmatics treated with LTMs. Nonetheless, some studies showed a significant influence of LTC4S-444A/C polymorphism on response to LTM. Thus, we performed an additional meta-analysis to evaluate the association between the SNP and lung function.

Study characteristics of the five studies included in the meta-analysis were shown in Table 3 and Table 4. One study that did not study the association between LT4CS-444A/C polymorphisms and lung function, five studies which had insufficient data to calculate the effect value, two studies that used adenosine monophosphate (AMP) and fractional concentration of exhaled nitric oxide (FENO) (the smaller the better, opposite to FEV_1_) as the outcome indicators of lung function, and one study that used HS required to cause a 20% drop in FEV1 (HS-PD20) as an indicator of LTMs response were excluded from the meta-analysis but are still described in our systematic review.

All studies included in our meta-analysis were cohort studies. All DNA samples studied were from blood samples. Among the five studies, two did not provide clear grades of asthma severity for the included patients; instead, they provided laboratory indicators for inclusion, which is equivalent to diagnostic criteria for asthma [23,24]. All studies investigated adults. Two studies examined asthmatics administered non-selective CysLT receptor antagonists, one administered pranlukast, and one took zafirlukast. The others were treated with montelukast 10 mg daily (selective CysLT1 receptor antagonists). Two studies excluded patients with AIA [23,24], two studies reported asthma subtypes that included AIA [22,25], and one study did not mention if patients with AIA were included [26]. Patients in four studies included in our meta-analysis did not receive oral corticosteroids (OCS) within a month prior to entering the origin study. Specifically, patients in Pan’s study did not take OCS or adjust ICS dosing for the last 2 months, and for 3 months in Asano’s study. In contrast, ten patients used OCS in Sampson’s study. Four studies included in our meta-analysis involved Asians [22,23,24,26], and the other was conducted in only Europeans [25]. Since some studies lacked patients with the mutant homozygous CC genotype, we compared all patients carrying C gene with the patients with AA genotype. The two studies that investigated LTC4S-444A/C polymorphism by AA vs. AC were Wu et al. and Pan et al. [24,26]. Most studies were assessed as having moderate or good quality based on NOS scores.

**Figure 2:**
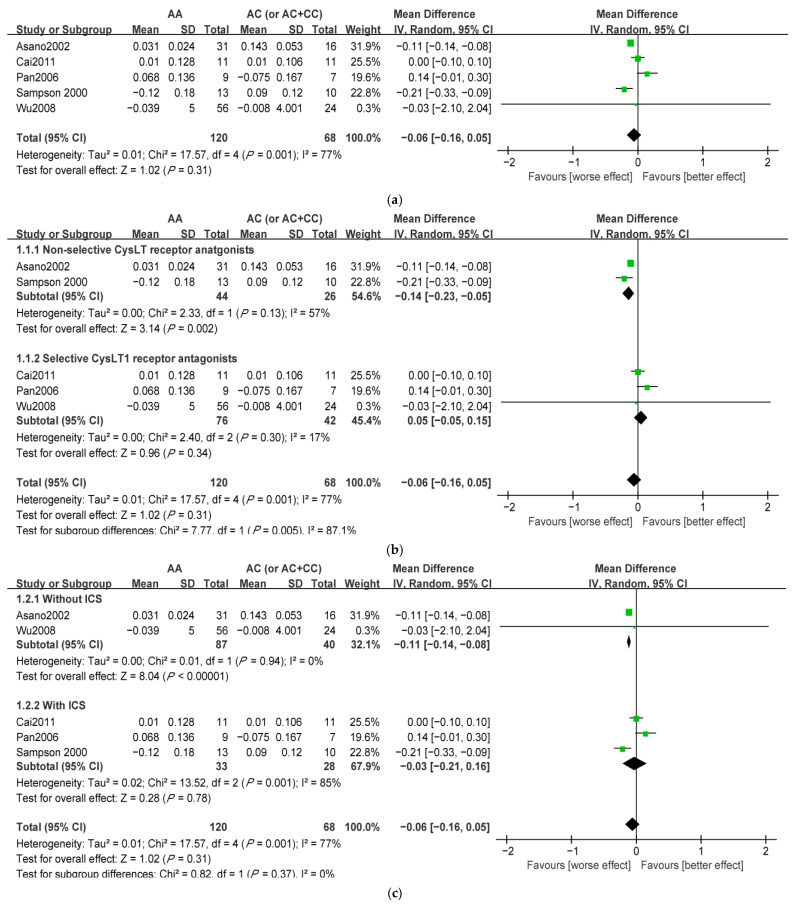
A meta-analysis was performed using random effects model for the association between improvement of lung function parameters and the LTC4S-444A/C polymorphism (AA vs. AC (or AC + CC)). (**a**) Ungrouped meta-analysis; (**b**) subgroup analysis for the types of drugs; (**c**) subgroup analysis for concomitant medication. The center of the green square represents the point estimate of the MD value of each study, and the size of the green square represents the weight of each study, the larger the weight the larger the square area. The length of the black line represents the range of 95% confidence intervals of the MD value for each study. The black diamond represents the combined effect of the meta-analysis for each study, the center of the diamond represents the MD value of the combined effect, and the width of the diamond represents the range of the 95% confidence interval of the combined effect.

Forest plots comparing MD value of LTC4S-444A/C genotype distributions between improvement of lung function parameters to LTMs are shown in Figure 2. Patients with AA genotype had no significant difference in improvement of lung function parameters compared with patients carrying C allele (MD = −0.06, 95%CI: −0.16 to 0.05, *p* = 0.31). Heterogeneity was observed between studies (I^2^ = 77%), implying that a subgroup analysis was needed to explore the heterogeneity. In subgroup analysis for types of drugs, the result in patients with non-selective CysLT receptor antagonists was significant (MD = −0.14, 95%CI: −0.23 to −0.05, *p* = 0.002; Figure 2b). Subgroup for patients without ICS showed a significant result (MD = −0.11, 95%CI: −0.14 to −0.08, *p* < 0.00001; Figure 2c). Subgroup analyses based on ethnicity, asthma severity, and follow-up time all showed no significant association between lung function and LTC4S (data not shown).

**Figure 3:**
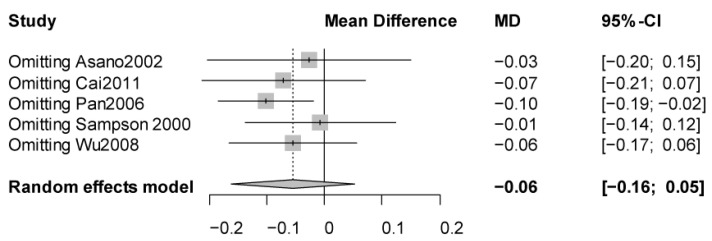
Sensitivity analysis of meta-analysis with a random effects model for the association between improvement of lung function parameters and the LTC4S-444A/C polymorphism (AA vs. AC (or AC + CC)).

None of the studies had a score of <5 on NOS. For sensitivity analyses, a significant association was observed when excluding Pan’s study (Figure 3). 

**Figure 4:**
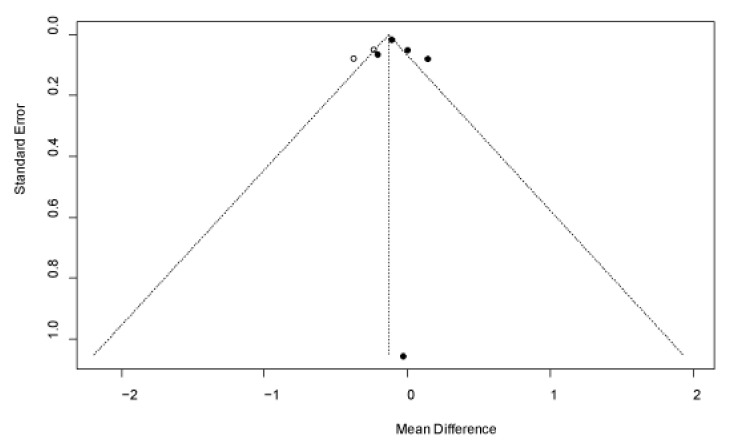
Begg’s funnel plot for publication bias of studies included in LT4CS-444A/C gene meta-analysis. The black dot represents the original study, the vertical line in the middle represents the MD value of the combined effect, and the diagonal areas on either side of the vertical line represent the 95% confidence intervals for the combined effect at different standard error scales. The white dot represents the new study that need to be added to correct the asymmetry of the funnel plot.

Publication bias was evaluated using Begg’s funnel plot (Figure 4) and Egger’s test. The Egger’s test suggested the absence of publication bias (t = 0.79, *p* = 0.4866), despite the seemingly asymmetric shape of the funnel plot.

Additionally, Asano et al. found a significant association between LTC4S-444A/C polymorphism and responsiveness to pranlukast [27]. In contrast, Kang et al. did not find a significantly different genotype distribution of LTC4S-444A/C in children who responded or did not respond to treatment, grouped by improvement of FEV_1_ [28]. According to the %change in FENO* (AUC_FENO_/Ƭ, Ƭ = the duration of the measurements) vs. time curve, Whelan et al. found that children with AC genotype had a more significant reduction than the AA genotype (*p* = 0.027) [29]. In addition, no significant association between LTC4S-444A/C and LTMs response was observed in other studies [20,30,31,32,33]. For asthma exacerbation, Lima et al. found that patients with heterozygotes had a 76% reduced risk compared with patients with AA homozygotes (*p* = 0.023) [13]. 

### 3.4. SLCO2B1 (rs12422149)

SLCO2B1 encodes organic anion transporting polypeptide 2B1 (OATP2B1), an organic anionic transporter that mediates sodium-dependent uptake of a variety of endogenous compounds, including leukotriene C4 [48]. Montelukast is absorbed through OATP2B1 and other possible transporters [34]. It was reasonable to speculate that SLCO2B1 gene affected the pharmacokinetics of montelukast by affecting OATP2B1 protein, and thus the efficacy of montelukast. Several studies have demonstrated that SLCO2B1 (rs12422149) exhibited a substantial effect on the pharmacokinetics of montelukast [29,36]. Mougey et al. conducted a retrospective analysis and a clinical trial in 2009 and 2011, which concluded that patients with AG genotype of SLCO2B1 (rs12422149) had lower montelukast plasma concentration [34,35]. Li et al. found similar results showing that montelukast clearance was higher in patients with AG and AA genotype (Table 5) [36]. However, Tapaninen et al. and Kim et al. did not find a significant relationship in healthy volunteers [49,50]. Hirvensalo et al. noticed that SLCO2B1 (rs73063122 and rs4149056) was associated with pharmacokinetics of montelukast’s metabolite in healthy volunteers [51].

### 3.5. Others

In addition, some studies focused on CysLTR1, CysLTR2, MRPP3, MLLT3, TBXA2R, CYP2C9, PLA2G4, PTGD2, and IL-13 (Table 6). Cysteinyl leukotrienes (CysLTs) bind to cysteinyl leukotriene receptor 1 (CysLTR1) and cysteinyl leukotriene receptor 2 (CysLTR2) to exert bronchoconstriction and other effects. Lee et al. (2007) and Kim et al. (2007) performed clinical trials on Korean patients with CysLTR1 927T>C; however, they observed no significant association with the drug response rate of montelukast [32,33]. In contrast, Kim et al. (2007) found a significant difference between patients keeping good control of asthma after a 6-month montelukast withdrawal and montelukast-dependent patients [33]. They also found that CysLTR1-634C>T was significantly associated with the drug response rate of montelukast both in 2 months and 1 year. One study investigated the influence of CysLTR2 (rs912277 and rs912278) on the efficacy of montelukast [20]. Klotsman et al. (2007) noticed that patients with C,C haplotype of CysLTR2 (rs912277 and rs912278) have better lung function (*p* = 0.02). 

Thromboxane A2 (TBXA2) also plays an important role in the development of asthma [8]. TBXA2 can also have a negative effect on LTC4 synthase activity [52,53]. It is reasonable to speculate that TBXA2 polymorphism would influence the response to montelukast. However, Kim et al. (2008) did not find an obvious association in Korean children carrying TBXA2R +795T>C or +924T>C with drug response, although a significant result was found when these two subgroups were aggregated [37]. 

Phospholipase A2 (PLA2) catalyzes the hydrolysis of membrane phospholipids to AA, implying that genes coding PLA2 might influence the process of asthma [7]. Rava et al. (2017) found that PLA2G4 gene polymorphism was a susceptible gene for adults with asthma [54]. However, Guo et al. (2019) did not find the significant influence of PLA2G4 (rs932476) on response to montelukast, despite the fact that a slightly better trend in AA genotype was observed [38]. 

Prostaglandin D2 (PGD2), the major cyclooxygenase metabolite of AA, produces an effect through the PGD2 receptor (PTGDR) [55]. Several studies found that the presence of PTGDR (−549, −441, and −197) polymorphisms might affect PTGDR expression [56,57,58]. Kang et al. (2011) found that C allele carriers of PTGD2-441T/C were likely to have poor responses to LTMs [28].

IL-13 induces Arachidonic Acid 15-Lipoxygenase (ALOX15) expression, an enzyme oxidizing polyunsaturated fatty acids, and causes eosinophil-mediated airway inflammation [59]. Some studies speculated that IL-13 could promote allergen-induced airway hyperresponsiveness [60]. However, no relationship between IL-13 –1512A/C, –1112C/T, and +2044G/A and asthma susceptibility in Korean children with exercise-induced bronchoconstriction (EIB) was detected in Kang’s study [39]. Nonetheless, they found that IL-13–1112C/T CC genotype was significantly associated with better responses to montelukast.

It has been proved that uridine diphosphate-glucuronosyltransferase (UGT) mediated glucuronidation takes part in LTMs clearance [61]. Mosteller et al. (2014) found that UGT1A1*28 and UGT1A3*2 were associated with the oral clearance of GSK2190915 (a FLAP inhibitor) [40]. Angiotensin II (Ang II) encoded by Angiotensinogen (AGT) gene, which is a potent bronchoconstrictor agent, has been found to be associated with the release of LTC4 and elevated in patients with acute asthma [62,63,64]. It is reasonable to speculate that there is a possible impact of AGT gene on LTMs response. Pasaje et al. (2011) found a significant association between AGT (+2401C>G and +2476C>T) and montelukast treatment response in AIA patients [41].

Two of the pharmacogenetics studies on LTMs were genome-wide association studies (GWAS) [42,43]. Dahlin et al. conducted GWAS in white and non-white patients in 2015 and 2016. In 2015, the Leukotriene Modifier or Corticosteroid or Corticosteroid-Salmeterol Trial (LOCCS) and Effectiveness of Low Dose Theophylline as Add On Therapy for the Treatment of Asthma (LODO) were used as discovery cohorts, and Characterizing the Response to a Leukotriene Receptor Antagonist and an Inhaled Corticosteroid (CLIC) and Pediatric Asthma Controller Trial (PACT) were used as replication cohorts [42]. None of the SNPs achieved genome-wide significance (*p* = 9.40 × 10^−8^) in LOCCS (discovery cohorts), and rs12659144 (*p* = 2.2 × 10^−6^), as the top-ranked SNP of 25 SNPs which approached genome-wide significance (*p* < 10^−5^), did not replicate in LODO (discovery cohorts) [42]. rs2247977 exceeded the threshold for genome-wide significance (*p* = 4.95 × 10^−8^) in LODO, though this result was not replicated in LOCCS. The 200 top-ranked SNPs from discovery cohorts were evaluated in CLIC and PACT (replication cohorts) and only four SNPs survived in the correction for multiple testing (combined *p* < 0.00025), including MLLT3 (rs6475448), WBSCR17 (rs7794356), rs953977, and rs1364805. Among these SNPs, MLLT3 (rs6475448) had a great influence on the response to montelukast. In the 2016 GWAS, Abbott trial 1 and Abbott trial 2 were used as discovery cohorts, and LOCCS and LODO were used as replication cohorts [43]. None of the SNPs achieved genome-wide significance in Abbott trial 1 and Abbott trial 2 (discovery cohorts). While combining *p*-values from discovery and replication cohorts, MRPP3 (rs12436663) achieved genome-wide significance [43]. In addition, while using LOCCS and LODO trials as replication analyses, GLT1D1 (rs517020) was replicated. This implies that GLT1D1 (rs517020) was associated with both zileuton and montelukast treatment responses.

## 4. Discussion

Asthma is a heterogeneous inflammatory disease, and genetic factors are a major contributor to asthma susceptibility and treatment response. Several studies have demonstrated that drug response to ICS might be associated with genetic factors, including CRHR1, GLCC1, FCER2, and TBX21 [65,66,67,68,69,70,71,72]. The association between ADRB2 and β_2_-adrenergic receptor agonists has become a popular topic in the study of asthma pharmacogenomics, focusing primarily on the improvement or asthma exacerbation after taking short-acting β_2_-agonists (SABA) or LABA [73,74,75,76,77]. However, few studies have investigated the association between gene polymorphism and therapeutic response of LTMs. The association of LTC4S-444A/C polymorphisms with LTMs response has been widely studied for its role on LTC4 synthase, the key enzyme in the generation of the proinflammatory mediators. To date, research on the association of LTC4S-444A/C with LTMs treatment response has not reached a consensus. Thus, we conducted a meta-analysis to clarify this association. Our study is the first meta-analysis that explores the association between LTC4S-444A/C polymorphism and improvement of lung function parameters after LTMs treatment. Considering that genetic characteristics, concomitant medication, asthma severity, types of drugs, and follow-up time may affect the results of meta-analysis, subgroup analyses were also performed by ethnicity, with or without ICS, disease status of asthma, selective CysLT1 receptor antagonists, and follow-up time. 

This meta-analysis included 120 patients with AA genotype and 68 patients with AC (or AC + CC) genotype to investigate the association between LTC4S-444A/C that codes LTC4S, and improvement of lung function parameters after LTMs treatment. Our results indicated that, according to improvement of lung function parameters and LTC4S-444A/C polymorphism, no significant association was found in asthmatics treated with LTMs (*p* = 0.31). However, in the subgroup of patients with CysLT1 and CysLT2 receptor antagonists, the wild-type AA homozygous showed poor responses to LTMs treatment compared with C allele carriers (*p* = 0.002). This result was not presented in the subgroup of patients with selective CysLT1 receptor antagonists. This suggests that non-selective CysLT receptor antagonists (e.g., pranlukast or zafirlukast) may be more easily affected by LTC4S polymorphism than selective CysLT1 receptor antagonists (e.g., montelukast). Montelukast, a selective and potent LTD4 receptor antagonist, inhibits the bronchoconstriction by LTD4 binding to the CysLT1 receptor, which is inactive for CysLT2 receptor binding [78]. Due to the extremely low activity of LTC4 and CysLT2 binding (the IC50 values of LTC4, LTE4, and LTD4 for CysLT2 receptor are 350, 200, and 0.9 nM, respectively), it cannot antagonize contractions by LTC4 and LTE4. However, zafirlukast not only antagonizes the CysLT1 receptor, but also weakly antagonizes the binding of LTD4 and CyLT2 receptors. Pranlukast has an activity for both CysLT1 and CysLT2 receptors and antagonizes LTC4, LTD4, and LTE4 [78,79]. Thus, when the receptor is saturated, zafirlukast and pranlukast are susceptible to the influence of LTC4S over-expression. The C allele may be associated with a higher expression of LTC4 synthase, with the finding of LTC4 synthase mRNA in peripheral blood eosinophils in AIA patients [80]. This assumption is consistent with our results showing that patients with C allele had better responses to CysLT1 and CysLT2 receptor antagonists, but not to selective CysLT1 receptor antagonists.

We also performed a subgroup analysis of concomitant medication. A significant effect was found in patients without ICS (*p* < 0.00001), but not in patients with ICS (*p* = 0.78). This result suggests that ICS may interfere with LTMs responses in patients with LTC4S-444A/C polymorphism. Corticosteroid has been shown to upregulate the transcriptional activity of LTC4S gene and subsequently the activity of the LTC4S enzyme [81]. The effect was even more pronounced in C carriers of LTC4S-444A/C polymorphism. Thus, it may affect the treatment response in C carriers, counteracting the influence of LTC4S-444A/C polymorphism. This may also explain why we observed significant effects in patients who did not take ICS.

In addition, subgroup analyses of the other possible factors did not show significant results. However, the subtype of asthma (whether AIA patients are included) and the group of different types of drugs overlap. This is one of the limitations of our meta-analysis, though patients with AIA made up only a small percentage (5.7%) and thus may not have significantly impacted our results. 

According to I^2^ statistic and χ^2^ test, there exists heterogeneity in our meta-analysis (I^2^ = 77%). Consequently, subgroup analysis was performed to investigate the heterogeneity. Heterogeneity was significantly reduced when we grouped by types of drugs (I^2^ = 57% and 17%) and concomitant medication (I^2^ = 0% and 85%), implying that that heterogeneity may have derived from the selectivity of LTMs and concomitant ICS. However, sensitivity analysis showed statistically significant results if Pan’s study was omitted, suggesting that our result might be a false negative. This is also one of the limitations of our meta-analysis. We suggest that the unreliability may be caused by the following reasons: (1) Pan’s study only investigated 18 patients; thus, its results and statistical power are affected by sample size; (2) lack of patients with CC genotype may increase the heterogeneity of the study; (3) follow-up time was too short to observe valid results; and (4) 18 of 102 patients in the cohort were selected for pharmacogenomics studies, but the reasons for the selection were not explained explicitly in original studies, which potentially introduced selection bias. Publication bias in this meta-analysis was assessed by Begg’s funnel plots and Egger’s test. No significant result was detected; therefore, publication bias is unlikely.

For nearly two decades, more than 50 researchers have studied how genetic factors affect asthma treatment, and more than 20 studies have investigated the role of gene polymorphism in response to LTMs. However, these findings are still inconclusive. To date, the most widely studied gene polymorphisms in pharmacogenomics of LTMs treatment response were ALOX5 (tandem repeats of the Sp1-binding domain and rs2115819), LTC4S-444A/C, and SLCO2B1 (rs12422149). We performed a meta-analysis to examine the specific association, and achieved a negative result. Despite the positive results obtained in subgroup analyses, we cannot draw a firm conclusion due to the limited number of available studies. It is worth noting that there are currently two GWAS studies in pharmacogenomics of LTMs treatment. MLLT3 (rs6475448), WBSCR17 (rs7794356), MRPP3 (rs12436663), and GLT1D1 (rs517020) approached or achieved replicated significance [42,43]. This provides a new direction for more specific candidate gene studies in the future.

In general, three different mechanisms are involved in the effect of gene polymorphisms on treatment response: (1) variations that affect drug metabolism, (2) variations associated with the mechanisms that lead to adverse reactions, and (3) variations in therapeutic targets or pathways activated by medications [82]. Our systematic review focused on the influence of drug metabolism and therapeutic targets or pathways, with less attention to adverse reaction. This is also one of the limitations of our review. Evidence on the pharmacogenomics aspects of LTM adverse reactions remains scant. Fortunately, the survey of Pharmacogenomics in Childhood Asthma (PiCA) Consortium supported future pharmacogenomic studies on adverse reactions in patients with asthma and prioritized the study of adverse reactions for each class of anti-asthmatic drugs [83,84]. More research may focus on these aspects in the future.

## 5. Conclusions

In conclusion, there is still great variability in the pharmacogenomics of LTMs treatment response, partly due to differences in study design, characteristics of included patients, and the limited number of available studies. Our meta-analysis and systematic review suggest that LTC4S-444A/C may influence the treatment response of patients taking non-selective CysLT receptor antagonists for asthma and patients taking LTMs not combined with ICS for asthma. However, the influence of LTC4S, ALOX5, and the other gene polymorphisms warrants further investigations.

## Figures and Tables

**Table 1:** Included pharmacogenomics studies of response to LTMs in systematic review.

Ref.	Main Gene and SNPs	Population	Sample Size	Outcome
[13]	ALOX5 (tandem repeats of the Sp1-binding domain), *ALOX5* (rs2115819), LTA4H (rs2660845), LTC4S-444A/C (rs730012), MRP1 (rs119774)	Adults	61	Asthma exacerbation rate. Changes in FEV_1_% pred.
[14]	ALOX5 (tandem repeats of the Sp1-binding domain)	Children	1297	USMA. Urinary leukotriene E4.
[15]	ALOX5 (tandem repeats of the Sp1-binding domain)	Children and adults	61	FEV_1_ in % of predicted. Need of rescue medication. Number of exacerbations.
[16]	ALOX5 (tandem repeats of the Sp1-binding domain)	Adults	52	FEV_1_. FEF_25–75_. PEFR. AMP PC_20_.
[17]	ALOX5 (tandem repeats of the Sp1-binding domain)	Adults	114	FEV_1_.
[18]	ALOX5 (rs2115819)	Children and adults	478	Changes in FEV_1_.
[19]	ALOX5 (rs2115819), LTA4H (rs2660845)	Adults	21	Changes in PEF#. Changes in FEV_1_. The subjective symptom scores.
[20]	ALOX5 (rs4987105 and rs4986832), LTC4S-444A/C (rs730012), CysLTR2 (rs912277 and rs912278)	Children and adults	166	Change in AM PEF. Changes in FEV_1_% pred.
[21]	LTA4H (rs2660845)	Children and adults	3594	At least one exacerbation event.
[22]	LTC4S-444A/C (rs730012)	Adults	47	Changes in FEV_1_% pred.
[23]	LTC4S-444A/C (rs730012), LTA4H (rs2660845), ALOX5 (rs2115819, rs4986832, rs4987105)	Adults	22	The distribution of genotypes in asthma group and control group. FEV_1_. FEV_1_/FVC (%). Urinary excretion of LTE_4_.
[24]	LTC4S-444A/C (rs730012)	Adults	16	FEV_1_ improvement.
[25]	LTC4S-444A/C (rs730012)	Adults	23	Changes in FEV_1_. Changes in FVC. Changes in PEF.
[26]	LTC4S-444A/C (rs730012)	Adults	80	FEV_1_% pred. ACQ. Urinary excretion of LTE_4_.
[27]	LTC4S-444A/C (rs730012)	Adults	50	FEV_1_ improvement.
[28]	LTC4S-444A/C (rs730012), PTGD2-441T/C	Children	92	Drug response rate.
[29]	LTC4S-444A/C (rs730012)	Children	12	%change in FENO *
[30]	LTC4S-444A/C (rs730012)	Children and adults	78	BHR to AMP or methacholine. Changes in FEV_1_. Exhaled NO. Peripheral blood eosinophils.
[31]	LTC4S-444A/C (rs730012)	Adults	37	Response to hypertonic saline inhalation.
[32]	LTC4S-444A/C (rs730012), CysLTR1 927T>C	Children	100	Drug response rate.
[33]	LTC4S-444A/C (rs730012), CysLTR1 927T>C, CysLTR1-634C>T	Adults	89	Drug response rate.
[34]	SLCO2B1(rs12422149)	Adults	80	Montelukast plasma concentration. ASTM ASUI.
[35]	SLCO2B1(rs12422149)	Adolescents and young adults	24	AUC_0→__∞_ and Cmax of montelukast.
[36]	SLCO2B1(rs12422149)	Children	50	Montelukast clearance.
[37]	TBXA2R +795T>C and TBXA2R +924T>C	Children	100	Drug response rate.
[38]	PLA2G4 (rs932476)	Children	128	Drug response rate.
[39]	IL-13–1112C/T, IL-13–1512A/C and IL-13 +2044G/ A	Children	53	Drug response rate.
[40]	UGT1A1*28 and UGT1A3*2	Children and adults	444	Oral clearance of GSK2190915.
[41]	AGT (+2401C>G and +2476C>T)	Adults	56	Drug response rate.
[42]	MLLT3 (rs6475448), WBSCR17 (rs7794356), rs953977 and rs1364805	Children and adults	133	Mean ΔFEV_1_ from baseline
[43]	MRPP3 (rs12436663) and GLT1D1 (rs517020)	Adults	526	Mean ΔFEV_1_ from baseline.

Asthma exacerbation rate: The binary risk of having an asthma exacerbation (no exacerbation or at least one exacerbation) during 6 months of montelukast treatment. Need of rescue medication: Number of puffs of short-acting β_2_-agonists (SABA) per day during the last month of 6 months. Number of exacerbations: Comparing with baseline, the number of times a decrease in PEFR in the morning of at least 25% occurred during the last month. FEV_1_: Forced expiratory volume in 1 s. FEF_25–75_: Forced mid-expiratory flow rate. PEFR: Peak expiratory flow rate. AMP PC_20_: Adenosine monophosphate 20% fall in forced expiratory volume in 1 s. USMA: The number of unscheduled medical attendances for wheezing episodes. Changes in FEV_1_: Change in FEV_1_ from baseline after LTM treatment. Changes in FEV_1_% pred: %Change in predicted FEV_1_ from baseline after LTM treatment. #: Changes in PEF: The difference between the peak expiratory flow (PEF) rate (L/min) recorded in the patient’s asthma diary within the 4 weeks before montelukast administration, and a ‘post-value’ recorded within 4–8 weeks (change (L/min) = ‘post-PEF’ − ‘pre-PEF’). The subjective symptom score: Using a four-point evaluation scale (0—worse, 1—unchanged, 2—improvement, 3—marked improvement) completed by the doctor after montelukast therapy. FEV_1_ improvement was calculated as follows: 100 × (FEV_1_post − FEV_1_pre)/FEV_1_pre. Changes in PEF: Change in PEF from baseline after LTM treatment. *: %change in FENO: AUC_FENO_/Ƭ, Ƭ = the duration of the measurements. BHR: Bronchial hyperresponsiveness. AMP: Adenosine monophosphate. ASTM ASUI: Arc-sine transformed mean Asthma Symptom Utility score. Drug response rate: Based on FEV_1_ improvement after leukotriene antagonists’ treatment, the drug response rate was defined as “responders” or “non-responders” by the set cutoff value. GSK2190915: A 5-lipoxygenase-activating protein inhibitor (FLAP inhibitor), inhibiting the production of LTB4 and other cysteinyl leukotrienes.

**Table 2:** ALOX5 gene pharmacogenomics studies of response to LTMs.

Gene and SNPs	Study (Author, Year of Publication, and Study Design)	Population (Sample Size, Race, Age Range, and Asthma Severity)	Follow-Up Time	Medication (Type, Dosage, Regimen, and Drug Combination)	Outcome	Result
ALOX5 (tandem repeats of the Sp1-binding domain)	Lima et al. (2006) [13] Clinical trial	61 White patients (age 40 ± 15 years). Mild to moderately severe persistent asthma that was not well controlled.	6 months	Montelukast 10 mg daily as add-on therapy, with/without ICS	Asthma exacerbation rate.	Patients carrying a variant number (either 2, 3, 4, 6, or 7) of repeats of the ALOX5 promoter on one allele had a 73% reduction in the risk of having at least one asthma exacerbation compared with homozygotes for the five repeat alleles (*p* = 0.045).
	Nwokoro et al. (2014) [14] Clinical trial	1297 children in England and Scotland (range 10-month from 5-year). Mild asthma ǂ.	12 months	Montelukast with/without ICS	USMA. Urinary leukotriene E4.	Comparing with placebo group, patients with 5/5 genotype had a reduction of USMA risk (IRR: 0.80, 95%CI: 0.68 to 0.95; *p* = 0.01) in montelukast group, while no difference was found in patients with 5/x + x/x genotypes. Patients with x/x genotype had higher urinary leukotriene E4 than 5/5 genotype (*p* = 0.02). However, there was no significant difference between 5/5 and 5/x genotypes, or 5/5 and 5/x + x/x genotypes.
	Telleria et al. (2008) [15] Clinical trial	61 patients (mean age 24.9 years, range 14–52). Moderate persistent asthma that was not well controlled.	6 months	Montelukast as add-on therapy, budesonide (500 mg/12 h) and β_2_ agonist used on demand	FEV_1_ in % of predicted. Need of rescue medication. Number of exacerbations.	Patients with at least one five repeat allele had lower exacerbations rate(4/5 + 5/5: 0.4 ± 0.21 with a mean reduction of 4.41 ± 2.76; 4/4: 1.88 ± 0.92 with a reduction of 1.33 ±1.22, *p* = 0.001), better FEV_1_ response (4/5 + 5/5: 93% ± 7.0 with an increase of 8.6% ± 7.61; 4/4: 80% with a reduction of 1.4% ± 8.04; *p* = 0.0006), and less β_2_ rescue medication (4/5 + 5/5: 2.9 ± 2.77 with a reduction of 4.3 ± 3.42; 4/4: 5.1 ± 2.84 with a reduction of 0.55 ± 1.66; *p* = 0.0011) than patients with 4/4 repeats allele.
	Fowler et al. (2002) [16] Retrospective analysis	52 patients (mean age 32–39 years). Mild to moderate atopic asthma.	1–2 weeks	Montelukast or zafirlukast, with/without ICS or oral anti-histamine	FEV_1_. FEF_25–75_. PEFR. AMP PC_20_.	In terms of FEV_1_, FEF_25–75_, PEFR, and AMP PC_20_, patients with LTMs or placebo had no significant difference in wild-type homozygotes and heterozygotes. However, it showed a non-significant trend of a greater increase in PEFR response in heterozygotes than wild-types (heterozygotes: 20 ± 9.2, wild-type: 10 ± 4.8; 95% CI: −38 to 1).
	Drazen et al. (1999) [17] Clinical trial	114 adults across the United States. Mild asthma ƚ.	2 weeks	ABT-761 300 mg/d with albuterol used on demand	FEV_1_.	Patients with the wild-type or heterozygous genotype had a significantly greater change in FEV_1_ than patients harboring the mutant genotype (18.8 ± 3.6%, 23.3 ± 6.0%, −1.2 ± 2.9%; *p* < 0.0001 and *p* = 0.0006).
ALOX5 (rs2115819)	Lima et al. (2006) [13] Clinical trial	61 White patients (age 40 ± 15 years). Mild to moderately severe persistent asthma that was not well controlled.	6 months	Montelukast 10 mg daily as add-on therapy with/without ICS	Changes in FEV_1_ % pred.	Patients with GG genotype had a significantly higher FEV_1_ response to montelukast compared with AA and AG genotypes (GG: 30%, 95%CI: −0.017 to 1.21; AA: 4.4%, 95%CI: −0.025 to 0.66; AG: 2.0%, 95%CI: 0.013 to 0.075).
	Tantisira et al. (2009) [18] Clinical trial	478 Caucasian (age 12-year or older). Moderate asthma.	12 weeks	zileuton CR, 1200 mg twice daily or zileuton IR, 600 mg 4 times daily with albuterol used on demand	Changes in FEV_1_.	Patients carrying G allele had higher changes in FEV_1_ than AA homozygous (*p* = 0.01).
	Kotani et al. (2012) [19] Clinical trial	21 Japanese adults. Mild to moderate persistent asthma *.	4–8 weeks	Montelukast 10 mg daily	Changes in PEF. Changes in FEV_1_ #. The subjective symptom scores.	Patients with GG homozygous had higher FEV_1-0_ than those carrying A allele before montelukast therapy (*p* < 0.05). There was no significant difference in change in PEF and FEV_1_ between GG homozygous and A allele carriers (*p* = 0.13 and 0.90). The subjective symptom score tended to be lower in GG homozygous (*p* = 0.052).
ALOX5 (rs4987105 and rs4986832)	Klotsman et al. (2007) [20] Retrospective analysis	166 patients (include Caucasian, Hispanic, African Americans, and others; age > 15 years) Moderate to severe persistent asthma ƚ	12 weeks	Montelukast 10 mg daily	Change in AM PEF	Patients with T,A haplotype had better improvement in AM PEF than patients with C,G haplotype of CysLTR2 (*p* = 0.003).

Asthma exacerbation rate: The binary risk of having an asthma exacerbation (no exacerbation or at least one exacerbation) during 6 months of montelukast treatment. Need of rescue medication: Number of puffs of short-acting β_2_-agonists (SABA) per day during the last month of 6 months. Number of exacerbations: Comparing with baseline, the number of times a decrease in PEFR in the morning of at least 25% occurred during the last month. FEV_1_: Forced expiratory volume in 1 s. FEF_25–75_: Forced mid-expiratory flow rate. PEFR: Peak expiratory flow rate. AMP PC_20_: Adenosine monophosphate 20% fall in forced expiratory volume in 1 s. USMA: The number of unscheduled medical attendances for wheezing episodes. Changes in FEV_1_% pred: %Change in predicted FEV_1_ from baseline after 6 months of montelukast treatment. Changes in PEF: The difference between the peak expiratory flow (PEF) rate (L/min) recorded in the patient’s asthma diary within the 4 weeks before montelukast administration, and a ‘post-value’ recorded within 4–8 weeks [change (L/min) = ‘post-PEF’ − ‘pre-PEF’]. The subjective symptom score: Using a four-point evaluation scale (0—worse, 1—unchanged, 2—improvement, 3—marked improvement) completed by the doctor after montelukast therapy. ƚ: Mild asthma that was clinically stable for at least 6 weeks while using inhaled medium-acting β_2_-agonists as sole asthma treatment, with a predictive value of FEV_1_ between 40–75% at enrollment, and no significant comorbidities. ǂ: Mild asthma: Patients had two or more previous episodes of wheeze, at least one of which was physician-confirmed, and at least one of which had happened in the preceding 3 months. *: Mild to moderate persistent asthma: Patients keep an asthma diary every day and do not have an asthma exacerbation requiring oral or intravenous injection of steroid drugs and his/her peak flow rate is 60–90% of the predicted value more than before 1 month of montelukast therapy. #: Changes in FEV_1_: The difference in reversibility of FEV_1-0_ between pre-intervention values obtained within the 4 weeks before montelukast administration and post-intervention values obtained within 4–8 weeks after the start of montelukast administration was calculated [changes (L) = post-FEV_1-0_ − pre-FEV_1-0_].

**Table 3:** Characteristics of studies included in LT4CS-444A/C gene meta-analysis.

Study/Ref.	Ethnicity	Participants (Number, Age)	Sex Ratio (Male/Total; %Male)	Asthma Severity	Medication	Dose of LTMs	Concomitant Medication	Asthma Subtype	Follow-Up Time
Asano 2002 [22]	Japanese	47 26–75 years ƚ	31/19 62% ƚ	Moderate asthma #	Pranlukast	225 mg twice daily	Salbutamol	One with aspirin sensitivity, others n/a	4 w
Cai 2011 [23]	Chinese Han	22 18–61 years ǂ	22/38 36.7% ǂ	Mild to moderate stable asthma ǂ	Montelukast	10 mg daily	With/without ICS, with/without Theophylline, and so on	None of allergic disease	4 w
Pan 2006 [24]	Chinese Han	16 54 ± 10.9 years	8/8 50%	Not specified Ƭ	Montelukast	10 mg daily	ICS with/without bronchodilators	No one had an aspirin sensitivity history	2 w
Sampson 2000 [25]	Patients in UK	23 25–76 years	6/17 26.1%	Severe asthma	Zafirlukast	20 mg twice daily	ICS and β_2_-agonists as required	Three with aspirin sensitivity	2 w
Wu 2008 [26]	Chinese Han	80 48.5 ± 8.9 years *	92/58 61.3% *	Mild to severe asthma *	Montelukast	10 mg daily	β_2_-agonists	n/a	4 w

n/a: not available. AIA: aspirin-intolerant asthma. ƚ: Cases included in our meta-analysis excluded the “lost to follow-up” of the original literature by the original author, so we use the characteristics of the whole participants instead of cases. #: According to GINA (2020 update), patients who had been controlled with step-3 treatment (low dose ICS-LABA, medium-dose ICS, or low-dose ICS + LTRA). Subjects included in the original literature were well controlled with moderate ICS (fluticasone 200–400 μg/day or beclomethasone 400–800 μg/day). ǂ: Cases included in our meta-analysis were selected from the participants of the original literature by the original author (reason unknown), so we used the characteristics of the whole participants instead of cases. Ƭ: The severity of the disease in the included patients was not specified in the original literature, but all patients showed FEV_1_% predicted > 50%. *: Cases included in our meta-analysis were selected randomly from the participants of the original literature by the original author, so we use the characteristics of the whole participants instead of cases.

**Table 4:** Genotype characteristics of studies included in LT4CS-444A/C gene meta-analysis.

Study/Ref.	Genotyping Method	HWE (*p*-Value of χ^2^ Test)	NOS Scores	Definition of Outcome	AA			AC (or AC + CC)		
					Mean	Sd	Total	Mean	Sd	Total
Asano 2002 [22]	PCR-RFLP	0.984	8	% Improvement of FEV_1_ ƚ	0.031	0.024	31	0.143	0.053	16
Cai 2011 [23]	MALDI-TOF	0.926	8	Change in FEV_1_/FVC (%)	0.010 #	0.128 ǂ	11	0.010 #	0.106 ǂ	11
Pan 2006 [24]	PCR-RFLP	0.534	7	% Improvement of FEV_1_ ƚ	0.068	0.136	9	−0.075	0.167	7
Sampson 2000 [25]	PCR-RFLP	0.489~0.895	5	% Improvement of FEV_1_ ƚ	−0.120	0.180	13	0.090	0.120	10
Wu 2008 [26]	PCR-RFLP	0.288	9	Change in FEV_1_%	−0.039 Ƭ	5.000 ǂ	56	−0.008 Ƭ	4.001 ǂ	24

NOS: Newcastle-Ottawa Scale is a ten-point scale. PCR-RFLP: PCR-Restriction Fragment Length Polymorphism. MALDI-TOF: Matrix-assisted laser desorption time of flight. ƚ: FEV_1_ improvement was calculated as follows: 100 × (FEV_1_post − FEV_1_pre)/FEV_1_pre. #: mean was calculated as follows: FEV_1_/FVC (%) post − FEV_1_/FVC (%) pre. ǂ: sd was calculated as follows: (sdpost^2^ + sdpre^2^)^1/2.^ Ƭ: mean was calculated as follows: FEV_1_% post − FEV_1_% pre.

**Table 5:** *SLCO2B1* gene pharmacogenomics studies of response to LTMs.

Gene and SNPs	Study (Author, Year of Publication, and Study Design)	Population (Sample Size, Race, Age Range, and Asthma Severity)	Follow-Up Time	Medication (Type, Dosage, Regimen, and Drug Combination)	Outcome	Result
SLCO2B1 (rs12422149)	Mougey et al. (2009) [34] Retrospective analysis	80 adult patients (including African Americans, Caucasian, and Hispanic) Moderate asthma.	1 month and 6 months	Montelukast 10 mg daily	Montelukast plasma concentration. ASTM ASUI. *	Patients with AG genotype had lower montelukast plasma concentration than patients with GG genotype at both 1 month and 6 months (*p* = 0.019 and 0.025). ASTM ASUI was significantly improved in subjects with GG genotype at both 1 month and 6 months in montelukast (*p* < 0.0001). Subjects with AG genotype showed no significant improvement in ASTM ASUI in the whole follow-time (*p* = 0.84).
	Mougey et al. (2011) [35] Clinical trial	24 adolescents and young adults (including African Americans and European Americans; aged 15–18 years) Mild asthma.	12 h	Montelukast 10 mg daily, with/without SABA, with/without LABA, with/without ICS	AUC_0→__∞_ and Cmax of montelukast.	Compared with GG genotype, AG genotype was significantly associated with lower AUC_0→__∞_ and Cmax of montelukast (AUC_0→__∞_, AG vs. GG, 1460 ± 340 ng·h·mL^−1^ vs. 2310 ± 820 ng·h·mL^−1^, *p* = 2.0 × 10^−5^).
	Li et al. (2019) [36] Clinical trial	50 Chinese children (age 6 months to 12 years) Asthma.	24.8 h	Montelukast 4 mg (0.5–5 years) and 5 mg (6–12 years) daily, with/without loratadine.	Montelukast clearance.	Montelukast clearance was higher in patients with GA and AA genotype compared with GG genotype (0.94 ± 0.26 versus 0.77 ±0.21, *p* = 0.020). The significant relationship still existed under multiple linear regression adjustment (*p* = 0.045).

* ASTM ASUI: Arc-sine transformed mean Asthma Symptom Utility score.

**Table 6:** The other gene pharmacogenomics studies of response to LTMs.

Gene and SNPs.	Study (Author, Year of Publication, and Study Design)	Population (Sample Size, Race, Age Range, and Asthma Severity)	Follow-Up Time	Medication (Type, Dosage, Regimen, and Drug Combination)	Outcome	Result
CysLTR1 927T>C	Lee et al. (2007) [32] Clinical trial	100 Korean children. Asthma with EIB.	8 weeks	Montelukast 5 mg daily with SABA used on demand	Drug response rate *	No significant difference in genotype distribution was found between groups that were divided by drug response rate (*p* = 0.192).
	Kim et al. (2007) [33] Clinical trial	89 Korean adults. Mild to moderate persistent asthma with AIA.	2 months or 1 year	Montelukast 10 mg daily, ICS, with SABA used on demand	Drug response rate *	There is no significant difference in genotype distribution between patients uncontrolled under montelukast 2 months intake and montelukast-dependent patients during 1-year follow-up (*p* = 0.060). An obvious difference between patients controlled well after 6-month montelukast withdrawal and montelukast-dependent patients was observed (*p* = 0.016).
CysLTR1-634C>T	Kim et al. (2007) [33] Clinical trial	89 Korean adults. Mild to moderate persistent asthma with AIA.	2 months or 1 year	Montelukast 10 mg daily, ICS, with SABA used on demand	Drug response rate *	A significant difference in genotype distribution between patients uncontrolled under montelukast 2 months intake and montelukast-dependent patients during 1-year follow-up was observed (*p* = 0.017) and between patients controlled well after 6-month montelukast withdrawal and montelukast-dependent patients (*p* = 0.007).
CysLTR2 (rs912277 and rs912278)	Klotsman et al. (2007) [20] Retrospective analysis	166 patients (include Caucasian, Hispanic, African Americans, and others; age > 15 years) Moderate to severe persistent asthma ƚ	12 weeks	Montelukast 10 mg daily	Change in AM PEF	Patients with C,C haplotype had better improvement in AM PEF than patients with common T,T and T,C haplotypes (*p* = 0.02).
TBXA2R +795T>C and TBXA2R +924T>C	Kim et al. (2008) [37] Clinical trial	100 Korean children. Atopic asthma and non-atopic asthma with EIB.	8 weeks	Montelukast 5 mg daily, with SABA occasionally	Drug response rate *	There is no significant difference in the genotype distribution of both TBXA2R +795T>C and TBXA2R +924T>C between responder and non-responder groups (*p* = 0.063 and 0.831). Patients with CT+CC/TT (+795/+924) had worse treatment response (OR: 3.67, 95%CI: 1.15 to 11.15, *p* = 0.041).
PLA2G4 (rs932476)	Guo et al. (2019) [38] Clinical trial	128 Chinese children (age 2–5 years) Mild to severe asthma.	2 months	Montelukast 4 mg daily, with SABA, used on-demand and other symptomatic treatments	Drug response rate *	Patients with the AA genotype tended to respond better than those with the GG genotype, but statistical significance was not reached (*p* = 0.222).
PTGD2-441T/C	Kang et al. (2011) [28] Clinical trial	92 Korean children Asthma.	8 weeks	Montelukast 5 mg daily	Drug response rate *	Patients with C allele heterozygous or homozygous of the PTGDR-441T/C polymorphism were higher in number in non-responder groups (*p* = 0.038).
IL-13–1112C/T, IL-13–1512A/C and IL-13 +2044G/ A	Kang et al. (2008) [39] Clinical trial	53 Korean children Asthma with EIB	8 weeks	Montelukast 5 mg daily	Drug response rate *	A significant difference in the genotype distribution of IL-13–1112C/T between responder and non-responder groups (*p* = 0.024), though this result was not for IL-13–1512A/C and +2044G/A (*p* = 0.139 and 0.346).
UGT1A1*28 and UGT1A3*2	Mosteller et al. (2014) [40] Clinical trial of cross-over design	444 patients (78% were non-Hispanic whites); 403 patients (non-Hispanic white)	n/a	GSK2190915 Ƭ	Oral clearance of GSK2190915	In 41 patients, UGT1A1*28 and UGT1A3*2 allele, which are in linkage disequilibrium, were significantly associated with oral clearance of GSK2190915 (*p* = 3.8 × 10^–4^ and 1.2 × 10^–5^). This result was not replicated in 403 non-Hispanic white patients.
AGT (+2401C>G and +2476C>T)	Pasaje et al. (2011) [41] Clinical trial	56 patients AIA	12 weeks	Montelukast 10 mg daily	Drug response rate *	The MAF frequency of AGT (+2401C>G and +2476C>T) is higher in non-responder groups after corrections for multiple testing (*p* = 0.0008–0.02).
MLLT3 (rs6475448), WBSCR17 (rs7794356), rs953977 and rs1364805	Dahlin et al. (2015) [42] GWAS (discovery and replication)	133 patients (including white and non-white)	8 weeks	LOCCCS: montelukast 10 mg daily; LODO: montelukast 5 or 10 mg daily; CLIC: montelukast 5–10 mg nightly depending on age; PACT: montelukast 5 mg daily ǂ	Mean ΔFEV_1_ from baseline	MLLT3 (rs6475448), WBSCR17 (rs7794356), rs953977 and rs1364805 met criteria (multiple testing, combined *p* < 0.0002) for replication cohorts. MLLT3 (rs6475448), WBSCR17 (rs7794356), and rs953977 also achieved (or approached) genome-wide significance (*p* = 4.95 × 10^−8^). Patients with homozygous for rs6475448 had significantly increased ΔFEV_1_ in discovery and replication cohorts.
MRPP3 (rs12436663) and GLT1D1 (rs517020)	Dahlin et al. (2016) [43] GWAS (discovery and replication)	526 adults (including white and non-white). Moderate persistent asthma	12 weeks	Abbott trial 1: zileuton CR 1200 mg twice daily or zileuton immediate-release 600 mg four times daily; Abbott trial 2: zileuton CR 1200 mg twice daily; LOCCCS: montelukast 10 mg daily; LODO: montelukast 5 or 10 mg daily #	Mean ΔFEV_1_ from baseline	Patients with AA homozygous of MRPP3 (rs12436663) had poor response to zileuton than patients with AG or GG genotype (*p* < 10^−8^; achieved genome-wide significance). GLT1D1 (rs517020) from the replicated zileuton GWAS results was replicated in LOCCS (combined *p*-value = 1.25 × 10^−7^). Patients with GLT1D1 (rs517020) had worsening responses to both zileuton and montelukast.

n/a: not available. AIA: Aspirin-intolerant asthma. MAF: Minor allele frequency. * Drug response rate: Based on FEV_1_ improvement after leukotriene antagonists’ treatment, the drug response rate was defined as “responders” or “non-responders” by the set cutoff value. Ƭ: GSK2190915: A 5-lipoxygenase-activating protein inhibitor (FLAP inhibitor), inhibiting the production of LTB4 and other cysteinyl leukotrienes. ƚ Criteria for inclusion of patients: (1) persistent asthma > 6 months; (2) FEV_1_ between 50% and 80%; (3) ≥15% reversibility within 30 min after albuterol (two puffs of 180 μg). ǂ: Discovery cohorts: LOCCS and LODO. Replication cohorts: CLIC and PACT. LOCCS: Leukotriene Modifier or Corticosteroid or Corticosteroid-Salmeterol Trial; LODO: Effectiveness of Low Dose Theophylline as Add On Therapy for the Treatment of Asthma. CLIC: Characterizing the Response to a Leukotriene Receptor Antagonist and an Inhaled Corticosteroid; PACT: Pediatric Asthma Controller Trial. #: Discovery cohorts: Abbott trial 1 and Abbott trial 2. Replication cohorts: LOCCS and LODO.

## Data Availability

Not applicable.

## Supplementary Materials

The following supporting information can be downloaded at: https://www.mdpi.com/article/10.3390/jpm12071068/s1, Table S1: PRISMA 2020 Checklist for the systematic review and meta-analysis.
